# The Analgesia Effect of Aucubin on CFA-Induced Inflammatory Pain by Inhibiting Glial Cells Activation-Mediated Inflammatory Response via Activating Mitophagy

**DOI:** 10.3390/ph16111545

**Published:** 2023-11-01

**Authors:** Dandan Yao, Yongjie Wang, Yeru Chen, Gang Chen

**Affiliations:** 1 Department of Anesthesiology, Sir Run Run Shaw Hospital, School of Medicine, Zhejiang University, Hangzhou 310058, China; 2 Department of Anesthesiology, School of Medicine, Shaoxing University, Shaoxing 312000, China; 3 Key Laboratory of Elemene Class Anti-Cancer Chinese Medicines, Engineering Laboratory of Development and Application of Traditional Chinese Medicines, Collaborative Innovation Center of Traditional Chinese Medicines of Zhejiang Province, Hangzhou Normal University, Hangzhou 311121, China

**Keywords:** aucubin, inflammation, pain, PINK1, mitophagy

## Abstract

Background: Inflammatory pain, characterized by sustained nociceptive hypersensitivity, represents one of the most prevalent conditions in both daily life and clinical settings. Aucubin, a natural plant iridoid glycoside, possesses potent biological effects, encompassing anti-inflammatory, antioxidant, and neuroprotective properties. However, its impact on inflammatory pain remains unclear. The aim of this study is to investigate the therapeutic effects and underlying mechanism of aucubin in addressing inflammatory pain induced by complete Freund’s adjuvant (CFA). Methods: The CFA-induced inflammatory pain model was employed to assess whether aucubin exerts analgesic effects and its potential mechanisms. Behavioral tests evaluated mechanical and thermal hyperalgesia as well as anxiety-like behaviors in mice. The activation of spinal glial cells and the expression of pro-inflammatory cytokines were examined to evaluate neuroinflammation. Additionally, RNA sequencing was utilized for the identification of differentially expressed genes (DEGs). Molecular biology experiments were conducted to determine the levels of the PINK1 gene and autophagy-related genes, along with PINK1 distribution in neural cells. Furthermore, mitophagy induced by carbonyl cyanide m-chlorophenylhydrazone (CCCP) was employed to examine the roles of PINK1 and mitophagy in pain processing. Results: Aucubin significantly ameliorated pain and anxiety-like behaviors induced by CFA in mice and reduced spinal inflammation. RNA sequencing indicated PINK1 as a pivotal gene, and aucubin treatment led to a significant downregulation of PINK1 expression. Further GO and KEGG analyses suggested the involvement of mitochondrial function in the therapeutic regulation of aucubin. Western blotting revealed that aucubin markedly decreased PINK1, Parkin, and p62 levels while increasing LC3B expression. Immunofluorescence showed the predominant co-localization of PINK1 with neuronal cells. Moreover, CCCP-induced mitophagy alleviated mechanical and thermal hyperalgesia caused by CFA and reversed CFA-induced mitochondrial dysfunction. Conclusions: In summary, our data suggest that aucubin effectively alleviates CFA-induced inflammatory pain, potentially through triggering the PINK1 pathway, promoting mitophagy, and suppressing inflammation. These results provide a novel theoretical foundation for addressing the treatment of inflammatory pain.

## 1. Introduction

Inflammatory pain is characterized by persistent nociceptive hypersensitivity, which includes both allodynia and hypersensitivity in the injured site and adjacent tissues, leading to an unpleasant sensory and emotional experience [1]. It affects at least 25% of the general population and results in a significant economic load on patients and healthcare systems worldwide [2]. Currently, the efficacy of most analgesics (including opioids, non-steroidal anti-inflammatory drugs, and anticonvulsants) in addressing chronic pain is limited, and can even lead to severe side effects [3]. Therefore, it is imperative to urgently investigate the specific molecular mechanisms that underlie the generation and persistence of chronic pain with diverse etiologies. Recently, increased attention has been devoted to the development of novel therapeutics for pain management, focusing on traditional medicinal herbs and dietary supplements within the realm of drug discovery.

In recent years, researchers have found that glial cells, especially microglia and astrocytes, are non-neuronal cells in the central nervous system (CNS) that provide support and maintain the overall function of neurons, playing a crucial role in the development and modulation of pain [4,5]. Microglia were activated during the initial disease phase, whereas astrocyte activation occurred in the subsequent sustaining phase. In response to injury or inflammation, glial cells undergo activation and release pro-inflammatory cytokines, including interleukin-1β (IL-1β), interleukin-6 (IL-6), and tumor necrosis factor-α (TNF-α). Pro-inflammatory cytokines serve as signaling molecules of the immune response that promote neuroinflammation. Activated glial cells release proinflammatory cytokines, amplifying pain transmission by activating and sensitizing neurons, and, reciprocally, activated neurons communicate with glial cells, fostering persistent inflammation and prolonged pain sensitization [6].

Mitophagy is a selective autophagy process that specifically eliminates impaired mitochondria from within cells, thus contributing to maintaining cellular homeostasis [7]. Multiple mitochondrial quality control pathways have been identified to mediate the degradation of misfolded mitochondrial proteins, mitochondrial fission/fusion, and the phagocytosis and degradation of damaged mitochondria (mitophagy) [8]. Dysfunctional mitophagy or defective mitochondrial biogenesis leads to an overall less efficient mitochondrial pool with damaged ATP production and enhanced mitochondrial ROS generation, potentially affecting sensory processing [9]. PTEN-induced protein kinase 1 (PINK1) is a neuroprotective protein that induces depolarization of the inner mitochondrial membrane as a response to mitochondrial damage, resulting in the accumulation of PINK1 on the outer mitochondrial membrane [10]. Studies have demonstrated that PINK1 stabilizes at the mitochondrial outer membrane upon mitochondrial damage and activates the Parkin ubiquitin ligase via phosphorylating Parkin and ubiquitin [11,12]. Parkin promotes the recruitment of the ubiquitin-binding adaptor SQSTM1/p62 (p62) and recruits ubiquitinated cargo to autophagosomes by binding to LC3, thereby mediating mitophagy [13]. Autophagy also plays a crucial role in maintaining a balanced inflammatory response due to its regulatory function [14]. Preclinical and clinical evidence suggests that modulation of mitochondrial function holds promise for alleviating or eliminating pain, providing possible advantages across a range of rheumatic diseases. [15].

**Figure 1:**
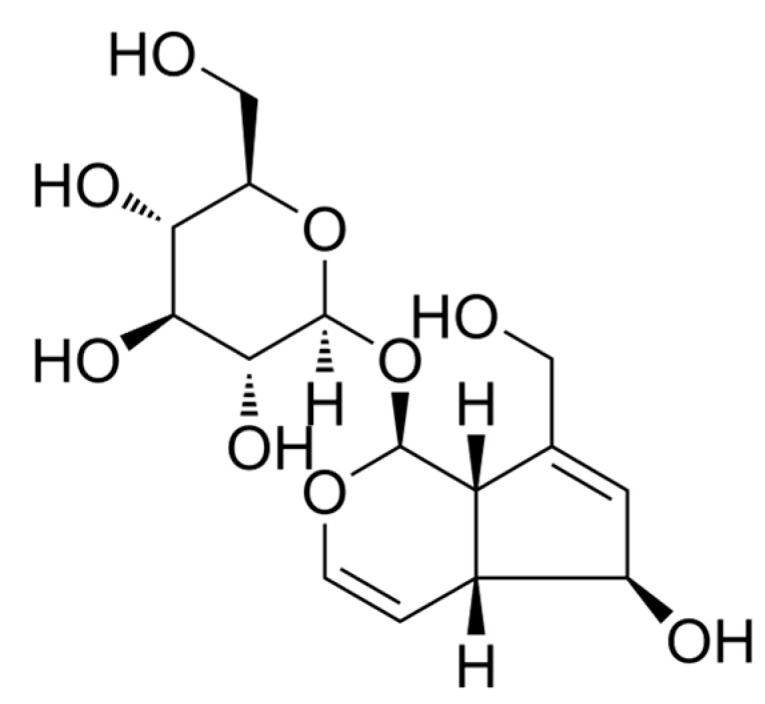
Chemical structure of aucubin.

Aucubin (Figure 1), an iridoid glucoside, is found in various plant species that exhibit potent antioxidant, anti-inflammatory, hepatoprotective, and neuroprotective effects [16]. Recent studies have demonstrated that aucubin effectively inhibits the activation of microglia and astrocytes, resulting in a significant inhibition of pro-inflammatory cytokines [17,18,19,20]. Furthermore, aucubin has been observed to elevate autophagy levels and inhibit apoptosis, thereby conferring neuronal protection [21,22]. Consequently, we formulated the hypothesis that aucubin could mitigate inflammatory pain by modulating the inflammatory response and promoting mitophagy. To validate this hypothesis, an inflammatory pain model was established in mice through intraplantar injection of complete Freund’s adjuvant (CFA), and subsequently, the role of aucubin on inflammatory pain was investigated.

## 2. Results

### 2.1. Effect of Aucubin on CFA-Induced Inflammatory Pain in Mice

**Figure 2:**
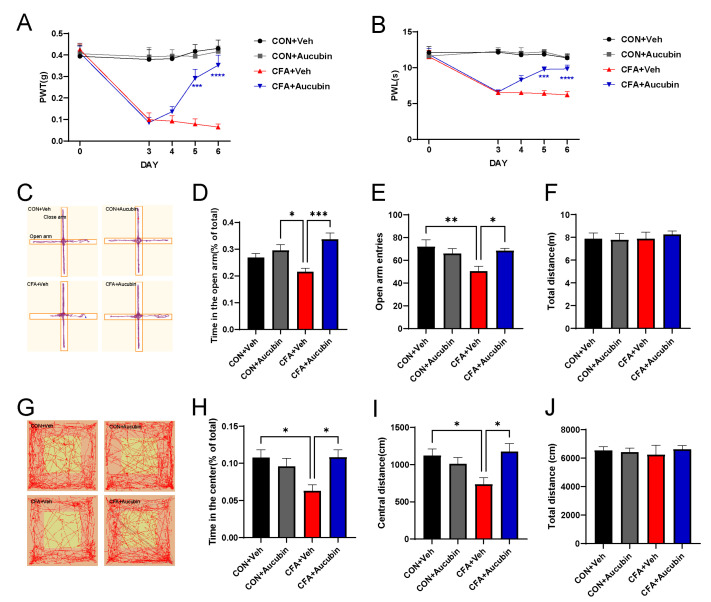
Effect of aucubin on CFA-induced inflammatory pain in mice. (**A**, **B**) Aucubin attenuates CFA-induced mechanical allodynia and thermal hyperalgesia in mice. Results are expressed as mean ± SEM (*n* = 8). *** *p* < 0.001, and **** *p* < 0.0001, CFA + Veh vs. CFA + Aucubin. Representative trajectories of locomotor activity in the EPM (**C**) and OFT (**G**). (**D**–**F**) Summarized data showed the time spent in the open arms, open arm entries, and total distance traveled in the EPM. (**H**–**J**) Summarized data showed the time spent in the central area, distance moved in the central area, and total distance moved in the OFT. Data are expressed as mean ± SEM (*n* = 8). * *p* < 0.05, ** *p* < 0.01, and *** *p* < 0.001.

We established an inflammatory pain model by injecting CFA into the left hindpaw of mice. Subsequently, we evaluated the effects of CFA-induced pain by detecting mechanical allodynia and thermal hypersensitivity. Behavioral tests revealed that the PWT and PWL in mice injected with CFA were significantly reduced compared to the control group (Figure 2A,B), indicating the successful induction of inflammatory pain. Remarkably, aucubin treatment significantly increased both PWT and PWL in CFA-injected mice (Figure 2A,B), suggesting its efficacy in alleviating mechanical allodynia and thermal hypersensitivity pain induced by CFA.

Chronic pain often co-occurs with psychiatric disorders, including anxiety, in clinical settings [23]. Thus, we employed the EPM and OFT to assess the anxiolytic effects of aucubin. CFA-treated mice exhibited a significant decrease in the number of entries into the open arms of the EPM, as well as a reduction in the time spent in the open arms, compared to the control mice (Figure 2C–E). Similarly, in the OFT, CFA-injected mice displayed reduced distance moved in the central area and a decreased time spent in the central area compared to the controls (Figure 2G–I). These results collectively indicate the presence of anxiety-like behavior in CFA-treated mice. In contrast, aucubin-treated mice demonstrated a significant increase in the time spent in the open arm and the number of open arm entries in the EPM (Figure 2C–E). Additionally, aucubin also increased the time spent in the center area and the distance moved in the central area of the OFT compared to the control group (Figure 2G–I). Crucially, the total distance traveled by the mice receiving either OFT or CFA did not show any discernible variations between the groups (Figure 2F,J), indicating that neither aucubin nor CFA affected the mice’s locomotor activity. Taken together, our results indicate that aucubin has the potential to alleviate pain and anxiety-like behavior in CFA-injected mice.

### 2.2. Effects of Aucubin on Inflammatory Responses in Mice Induced by CFA

**Figure 3:**
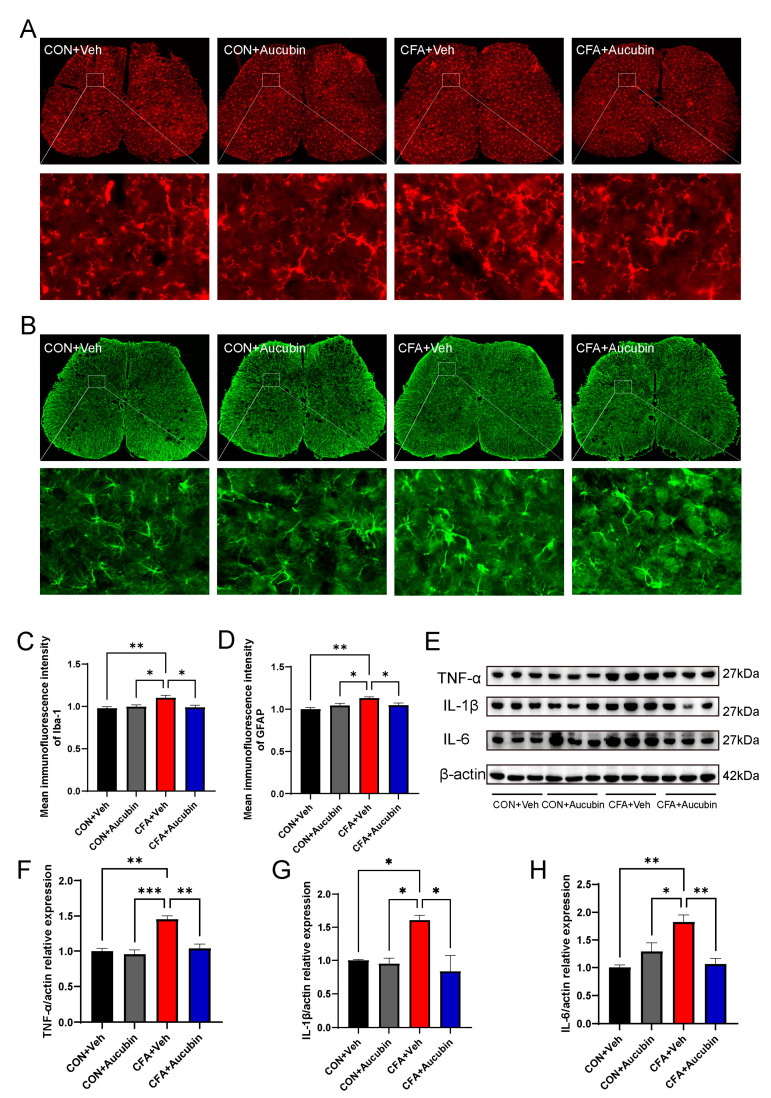
Effect of aucubin on CFA-induced glial cell activation and proinflammatory cytokine production in the spinal cord. (**A**,**B**) Representative immunofluorescence staining for microglia and astrocytes in the spinal dorsal cord of mice. (**C**,**D**) Mean immunofluorescence intensity of Iba-1 and GFAP (*n* = 3). Scale bars = 200 μm and 20 μm (magnification). (**E**–**H**) Representative bands of Western blot and quantitative analysis of the relative expression of TNF-α, IL-1β, and IL-6 (*n* = 3). Data are presented as mean ± SEM. * *p* < 0.05, ** *p* < 0.01, and *** *p* < 0.001.

To assess the effect of aucubin on inflammatory responses triggered by CFA, we utilized immunofluorescence and Western blot to examine its effect on the spinal glial cells and proinflammatory cytokines in mice. Specifically, we employed Ionized Calcium-Binding Adapter Molecule 1 (Iba-1) and Glial Fibrillary Acidic Protein (GFAP) as specific markers for astrocytes and microglia, respectively. As shown in Figure 3A–D, our results indicated that the intensity of Iba-1 and GFAP in the spinal dorsal horn of mice in the CFA group was dramatically increased compared to the control group, whereas treatment with aucubin (10 mg/kg) resulted in a noteworthy reduction of GFAP and Iba-1 expression.

Furthermore, previous study has demonstrated that activated astrocytes and microglia in the spinal cord produce and release a considerable amount of proinflammatory cytokines, which may directly sensitize nociceptive sensory neurons, thereby leading to pain [24]. Consistent with this, in the CFA group, the levels of IL-1β, IL-6, and TNF-α exhibited a marked elevation in comparison to the control group (Figure 3E–H). Conversely, aucubin treatment led to a notable decrease in the expression of IL-1β, IL-6, and TNF-α in CFA-injected mice (Figure 3E–H).

These results indicate that aucubin effectively alleviates CFA-induced inflammatory responses in the spinal cord.

### 2.3. RNA-Seq Analysis in the Spinal Cord of CFA-Injected Mice Treated with Aucubin

**Figure 4:**
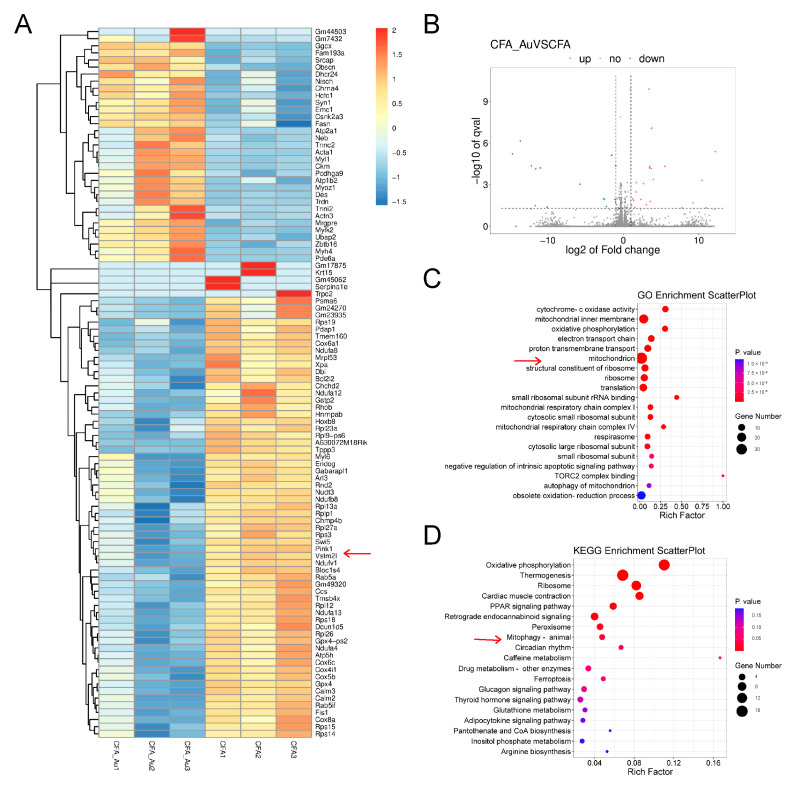
RNA−Seq analysis in the spinal cord of CFA−injected mice treated with aucubin. (**A**) Heatmap analysis of DEGs in CFA + Veh and CFA + Aucubin groups. The *x*−axis represents different groups, while the *y*−axis represents the DEGs. Upregulated genes are depicted in red, and downregulated genes are represented in blue. The arrow points to PINK1 gene. (**B**) Volcano plot of DEGs. Red indicates upregulated DEGs, blue represents downregulated DEGs, and grey represents non-significant DEGs. (**C**) GO enrichment analysis. (**D**) KEGG pathway analysis. The size of the circles corresponds to the number of DEGs, and the color gradient from red to blue indicates decreasing significance. The arrow points to mitophagy pathway.

To further investigate the potential mechanism by which aucubin alleviates inflammatory pain induced by CFA, we employed RNA sequencing to identify differentially expressed genes (DEGs) in CFA-injected mice with and without aucubin treatment. By analyzing the q-values, we identified the top 100 DEGs that significantly distinguished the two groups, as indicated by the heatmap (Figure 4A). The volcano plot displayed 22 upregulated DEGs and 15 downregulated DEGs in the spinal cord of CFA-injected mice after 3 days of aucubin treatment (Figure 4B), with upregulated genes shown in red and downregulated genes in blue. Moreover, the Gene Ontology (GO) enrichment analysis of DEGs demonstrated that the DEGs within the CFA + Aucubin group were primarily associated with mitochondrion, mitochondrial inner membrane, cytochrome C oxidase activity, oxidative phosphorylation, electron transport chain, and proton transmembrane transport when compared to CFA + Veh group (Figure 4C). The Kyoto Encyclopedia of Genes and Genomes (KEGG) pathway enrichment analysis indicated that the DEGs were significantly enriched in oxidative phosphorylation, thermogenesis, ribosome function, myocardial contraction, PPAR signaling pathway, and mitophagy (Figure 4D). Collectively, these results suggest that aucubin may mitigate CFA-induced inflammatory pain by modulating mitochondrial function.

### 2.4. Effects of Aucubin on Mitophagy in CFA-Injected Mice

**Figure 5:**
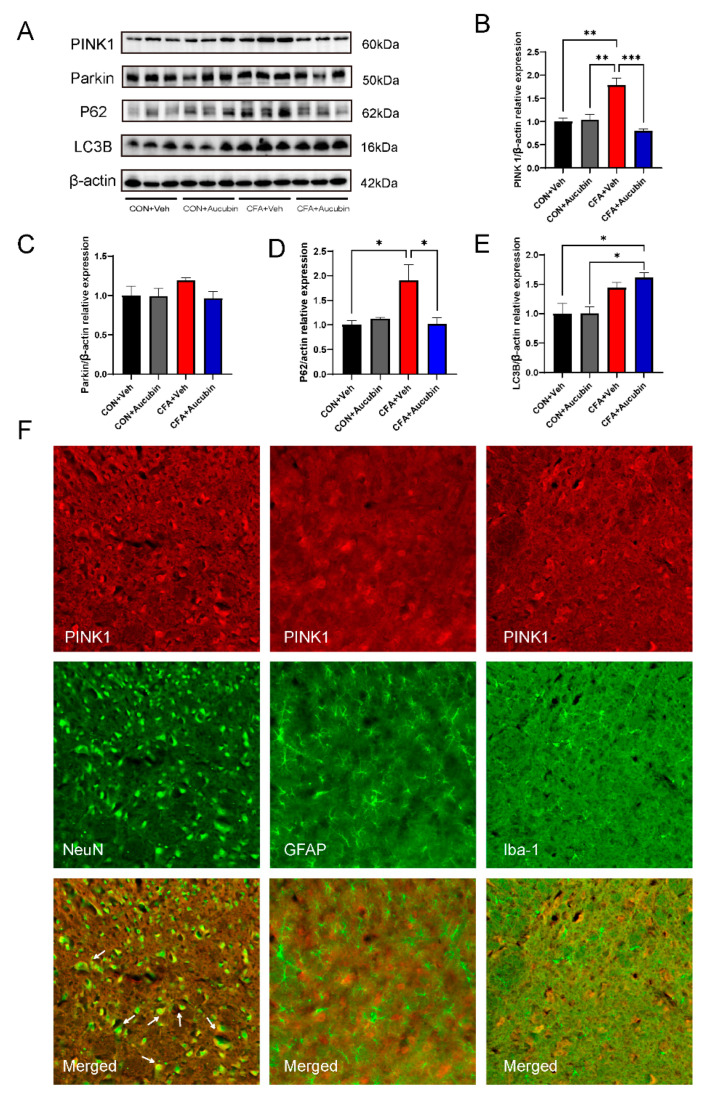
Effects of aucubin on mitophagy in CFA-injected mice. (**A**–**E**) Representative bands of Western blot and quantitative analysis of the relative expression of PINK1, Parkin, LC3B, and p62. Data are expressed as the mean ± SEM (*n* = 3). * *p* < 0.05, ** *p* < 0.01 and *** *p* < 0.01. (**F**) Double immunofluorescence staining of Neun (green), Iba-1 (green), GFAP (green) with PINK1 (red) after CFA injection for 6 days. Scale bar = 50 μm.

The PINK1 gene is closely associated with mitochondrial function and integrity, playing a vital role in activating mitophagy, a process linked to intracellular mitochondrial homeostasis and cell survival [25]. To explore the impact of aucubin on the PINK1 pathway, we examined spinal cord tissue from a mouse model of CFA-induced inflammatory pain using Western blotting and immunofluorescence. First, we assessed the expression levels of PINK1 and Parkin proteins of mice (Figure 5A). The results showed that the expression of PINK1 and Parkin was elevated after CFA injection compared to the control group (Figure 5B,C). Conversely, treatment with aucubin resulted in a decrease in PINK1 and Parkin expression compared to the CFA group (Figure 5B,C).

We then evaluated the expression of LC3B and p62 proteins through Western blotting. LC3B is a mammalian autophagy protein that localizes to the membrane of cytoplasmic autophagosome, while p62 serves as a well-known autophagic substrate degraded by LC3B and ubiquitinated substrates [13]. The accumulation of p62 indicates autophagic impairment, suggesting that autophagic flux is inhibited. Notably, a recent study has confirmed that autophagic degradation is blocked in osteoarthritis [26]. Consistent with this, CFA-injected mice exhibited increased expression of LC3B and p62 proteins (Figure 5D–E), indicating impaired autophagy in the spinal cord. Remarkably, aucubin treatment resulted in an increased LC3B expression and a decreased p62 expression compared to CFA-treated mice (Figure 5D–E), indicating that aucubin could ameliorate the impaired autophagic flow induced by CFA.

To further explore the cellular localization of PINK1, we conducted double immunofluorescence using three cell markers: NeuN, GFAP, and Iba-1. As shown in Figure 5F, the findings demonstrated that PINK1 exhibited co-localization with neurons, but not with microglia and astrocytes, in the spinal cord. Collectively, these results suggest that PINK1 is predominantly expressed in neuronal cells and that mitophagy in neuronal cells is involved in the pain process.

### 2.5. CCCP Significantly Alleviated CFA-Induced Inflammatory Pain in Mice by Activating Mitophagy

**Figure 6:**
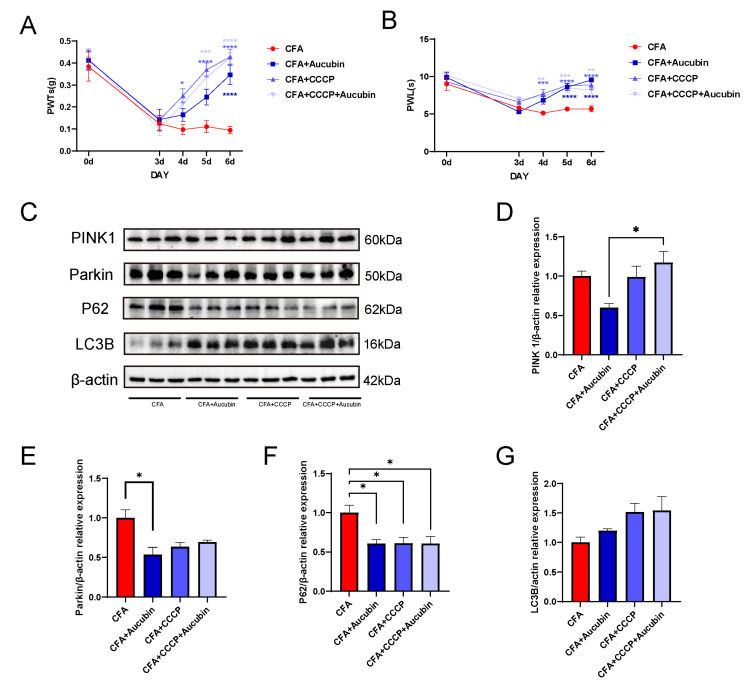
CCCP significantly alleviated CFA-induced inflammatory pain in mice by activating mitophagy. (**A**,**B**) CCCP attenuates mechanical allodynia and thermal hypersensitivity induced by CFA in mice (*n* = 9). (**C**–**G**) Representative bands of Western blot and quantitative analysis of the relative expression of PINK 1, Parkin, LC3B and p62 (*n* = 3). Data are expressed as mean ± SEM. * *p* < 0.05, ** *p* < 0.01, *** *p* < 0.001 and **** *p* < 0.001.

To further investigate the correlation between mitophagy and inflammatory pain, we employed CCCP as a pharmacological inducer of mitophagy. Mitophagy plays a vital role in the timely elimination of damaged mitochondria, but excessive autophagy activation may also lead to mitochondrial damage. Acting as a proton-selective ionophore, CCCP induces PINK1 accumulation, thus triggering mitophagy [27]. Previous study has showed that CCCP administration results in a dose-dependent increase in PINK1 and LC3B expression, effectively alleviating pain in a neuropathic pain model [28]. As illustrated in Figure 6A,B, there was a significant reduction in PWT and PWL in mice following CFA injection compared with baseline values on day 0. However, after CCCP treatment, we observed an upward trend in PWT and PWL, strongly suggesting that CCCP effectively reverses CFA-induced mechanical pain and thermal hypersensitivity.

Subsequently, we performed Western blotting to assess the protein expression of PINK1, Parkin, LC3B, and p62 in the spinal cord (Figure 6C). The results demonstrated that CCCP treatment led to a tendency of increased PINK1 expression and decreased Parkin expression compared with the CFA group (Figure 6D–E), indicating the effective activation of mitophagy by CCCP. Moreover, in the CCCP-treated groups, there was an increase in LC3B expression and a decrease in p62 levels compared to the CFA group (Figure 6F,G). These findings indicate that CCCP effectively restored the impaired autophagic flow in the spinal cord of mice subjected to CFA-induced inflammatory pain.

## 3. Discussion

The present study endeavors to elucidate the effects and underlying mechanisms of aucubin on inflammatory pain induced by CFA in mice. Our findings demonstrate that aucubin effectively reduces CFA-induced inflammatory pain and anxiety-like behavior. Furthermore, subsequent experiments revealed that aucubin exerts inhibitory effects on the activation of spinal astrocytes and microglia in CFA-injected mice, concomitantly reducing the expression of pro-inflammatory cytokines. Interestingly, aucubin also exhibits the ability to reverse the impaired autophagic flux induced by CFA injection and activate mitophagy. Taken together, our research results indicate that aucubin exhibits potential as a therapeutic avenue for ameliorating pain and neuroinflammation. These results offer a critical theoretical foundation for the continued advancement of relevant treatment approaches.

Mounting evidence highlights the pivotal role of neuroinflammation, characterized by glial cell activation and pro-inflammatory cytokine expression, in the establishment and persistence of central sensitization and pain [29]. Notably, microglia, as resident immune cells in the CNS, and astrocytes, with their star-shaped morphology and role in neuronal regulation, are key players in this process. Upon activation, microglia release pro-inflammatory cytokines that further stimulate astrocytes, initiating a cascade of inflammatory processes. Conversely, activated astrocytes can produce additional pro-inflammatory cytokines, resulting in the activation of glial cells and neurons, ultimately establishing a neuro-glial positive feedback loop, resulting in the sustained release of pain mediators [30].

Autophagy serves as a lysosomal degradation process responsible for clearing damaged proteins and organelles to maintain cellular homeostasis. It holds a crucial function in neuronal protection, as defects in autophagy or mitophagy are often linked to neuronal loss and cognitive decline in aging or CNS neurodegenerative disorders [31]. Mitophagy specifically governs the turnover of mitochondria, eliminating damaged ones, thus being essential in regulating mitochondrial quality and maintaining mitochondrial homeostasis. The outcome of mitophagy, whether beneficial or harmful to cell survival, depends on the level of mitophagy activation [32]. A pivotal pathway mediating mitophagy is the PINK1/Parkin pathway. Normally, PINK1 levels remain low under steady-state conditions, but upon mitochondrial damage, the PINK1 pathway becomes activated, causing the accumulation of PINK1 on the outer mitochondrial membrane and the recruitment of Parkin to damaged mitochondria [33].

A previous study has shown that models of osteoarthritis exhibit an augmentation in the expression of PINK1 and Parkin. [34]. Furthermore, studies have indicated that PINK1 expression is selectively induced in spinal dorsal horn neurons during neuropathic pain, leading to abnormal mitochondrial flux [35,36]. Consistent with these findings, our study demonstrates an upregulation of PINK1 and Parkin expression in CFA-injected mice, which was subsequently reversed by aucubin treatment. The potential mechanism underlying this effect might be attributed to aucubin promoting smooth mitophagy flow, resulting in the degradation of PINK1 anchored to the damaged mitochondrial outer membrane, consequently reducing the number of damaged mitochondria.

LC3B serves as a reliable marker of autophagic activity, and its upregulation can suggest enhanced autophagic flux as well as defective clearance of autophagosomes [37]. Additionally, we investigated p62, one of the autophagy-specific substrates that binds to LC3 and facilitates the recognition of damaged mitochondria by autophagosomes, aiding in their delivery to lysosomes for degradation [13]. Indeed, increased expression of p62 has been observed under autophagy impairment [38]. Previous study has shown that CFA administration led to elevated level of LC3B and p62 accumulation in the spinal cord, indicating blocked autophagic flux [39]. Similarly, our research demonstrates that in the CFA group, LC3B levels are elevated and accompanied by significant p62 accumulation. However, aucubin treatment significantly enhances LC3B levels and reduces P62 content, further activating mitophagy and restoring the blocked autophagic flux.

The relationship between autophagy and pain has been extensively investigated. Studies have indicated that impaired autophagy in glial cells in neuropathic pain models can lead to the secretion of pro-inflammatory cytokines, thereby exacerbating mechanical allodynia and thermal hyperalgesia [40,41]. Conversely, rapamycin has shown the ability to alleviate allodynia, hyperalgesia, and glial cell activation by inducing autophagy. Furthermore, increasing autophagy levels in neuronal cells have also been found to suppress the neuroinflammatory response and alleviate pain [42,43]. In our research, we demonstrated that the activation of mitophagy by CCCP effectively mitigated CFA-induced inflammatory pain. Similar studies have indicated that CCCP-induced activation of mitophagy also reduces pain sensitivity in neuropathic pain model [28]. This evidence suggests that mitophagy holds significant potential for future molecular pain research.

## 4. Materials and Methods

### 4.1. Chemicals and Reagents

Complete Freund’s adjuvant (CFA) was acquired from Sigma (St. Louis, MO, USA). Aucubin (HY-N0664) and carbonyl cyanide m-chlorophenylhydrazone (CCCP, HY-100941) were obtained from MedChemExpress (St. Louis, MO, USA). RIPA buffer (Catalog No.: HY-K1001) and PMSF (CAS No.: 329-98-6) were procured from MedChemExpress. Primary antibodies against the following proteins were obtained from ABclonal Technology (Wuhan, China): PINK1, TNF-α, IL-1β, IL-6, and β-actin (designated as A7131, A11534, A16288, A2447, and AC026, respectively). Additionally, primary antibodies against Iba-1 (#17198), GFAP (#3670), SQSTM1/p62 (#5114), and LC3B (#2775) were obtained from Cell Signaling Technology Company (Danvers, MA, USA). Antibodies against Iba-1 (ab283319) and NeuN (ab104224) were purchased from Abcam (Cambridge, MA, USA), while PINK1 (sc-517353) and Parkin (sc-32282) antibodies were obtained from Santa Cruz Biotechnology (Santa Cruz, CA, USA). Antibodies against Iba-1 (ab283319) and NeuN (ab104224) were purchased from Abcam (Cambridge, MA, USA), while PINK1 (sc-517353) and Parkin (sc-32282) antibodies were obtained from Santa Cruz Biotechnology (Santa Cruz, CA, USA). Fluorescently labeled secondary antibodies (ab150105 and ab150064) were procured from Abcam (Cambridge, MA, USA), and HRP-conjugated secondary antibodies (HA1001 and HA1006) were obtained from HuaBio (Hangzhou, China). Enhanced chemiluminescent solution (ECL) was obtained from Absin Biotechnology (Shanghai, China). The reagents of the TransZol Up Plus RNA Kit (ER501) used in the study were acquired from Transgene Biotechnology (Beijing, China).

### 4.2. Animals

Male C57BL/6 mice, aged 8–10 weeks and weighing 20–30 g, were obtained from the Zhejiang University Laboratory Animal Center. The mice were kept in standard laboratory conditions with free access to food and water. Prior to the behavioral tests, the mice were given about one week to acclimate to the laboratory environment. All necessary measures were taken to minimize the usage of animals and to ensure their ethical treatment and welfare during the study. Special attention was given to reducing any potential suffering experienced by the animals.

### 4.3. Experimental Protocol and Treatment Schedule

Inflammatory pain was induced by subcutaneously injecting 10 μL of CFA into the plantar surface of the left hindpaw of the mice, whereas the control group was given an equal volume of sterile saline in the hindpaw.

**Figure 7:**
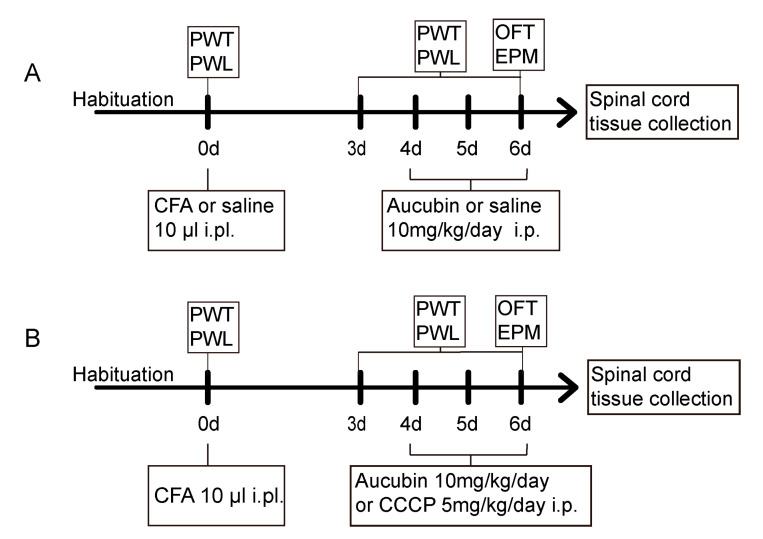
(**A**,**B**) Schematic representation of the experimental protocols.

Mice were allocated randomly into four groups to examine the anti-nociceptive effect of aucubin on CFA-induced inflammatory pain: Con + Veh, Con + Aucubin, CFA + Veh and CFA + Aucubin. After 3 days following the saline/CFA injection, mice received an intraperitoneal (i.p.) administration of 10 mg/kg aucubin or normal saline for three consecutive days, as illustrated in Figure 7A.

Mice were assigned randomly to four groups to further validate whether the analgesia effect of aucubin was mediated by activating mitophagy: CFA, CFA + Aucubin, CFA + CCCP and CFA + CCCP + Aucubin. It has been previously shown that CCCP induces the activation of PINK1 and promotes Parkin Ser65 phosphorylation, thereby triggering mitophagy. After the establishment of inflammatory pain induced by CFA, aucubin (10 mg/kg) or CCCP (5 mg/kg) was administrated i.p. from day 4 to day 6, as indicated in Figure 7B.

Throughout the experiment, the behavioral tests were conducted at a fixed time during the testing days, and the animals were allowed a 30 min habituation period in the testing room before the behavioral tests. Following the completion of the behavioral tests, the animals were sacrificed, and the L4-5 spinal cord segments were harvested for further processing and analysis.

### 4.4. Mechanical Allodynia

Mechanical allodynia was evaluated using the von Frey filaments following the up-down method as described by Chaplan [44]. Mice were placed on an elevated mesh platform and granted a 30 min habituation period before commencement of the test. Mechanical stimuli were applied to the middle of the plantar surface of each paw by calibrated von Frey filaments. The withdrawal thresholds were computed and documented as the paw withdrawal threshold (PWT).

### 4.5. Thermal Hyperalgesia

Thermal hyperalgesia was evaluated by the Hargreaves test [45]. Mice were placed individually on a raised box positioned on a glass plate, and a radiant heat source was positioned beneath the hind paw. The heat stimulus was applied to the plantar surface of the hind paw, and an automated timer was employed to measure the paw withdrawal latency (PWL). The test was repeated three times, with a 5 min interval between each repetition, and subsequently, the average PWL was calculated. To prevent tissue damage, a 20 s cut-off time was implemented, after which the heat stimulus was automatically terminated.

### 4.6. Elevated Plus Maze Test

The elevated plus maze (EPM) test was performed as previously established protocol [46]. The maze was composed of two open arms and two closed arms, elevated above the floor. Mice were individually placed at the center of the maze, oriented toward one of the open arms, and their behavior was recorded for a 5 min period. Prior to testing the next mouse, the maze was cleaned with 75% ethanol. Movements of the mice were tracked and quantified by ANY-maze 6.32 software (Stoelting, Wood Dale, IL 60191, USA).

### 4.7. Open Field Test

The open field test (OFT) was performed following previously described methods [47]. Prior to the experiments, mice were habituated to the testing condition and light level for 30 min. Mice were placed individually in a square arena (dimensions: 45 × 45 × 45 cm^3^) and allowed to explore freely for a duration of 15 min. After each test, the open field arena was cleansed using 75% ethanol. During the test, locomotor and exploratory behaviors of the mice were recorded through ANY-maze software.

### 4.8. Immunofluorescence Staining

Mice were anesthetized by intraperitoneal administration of sodium pentobarbital (50 mg/kg), followed by transcardial perfusion using phosphate-buffered saline (PBS) and subsequently 4% paraformaldehyde (PFA). Tissue sections were prepared from the spinal cord L4-5, fixed in PFA overnight at 4 °C, and subsequently permeabilized with 30% sucrose until saturation. Samples were freeze-mounted in OCT compound and sliced into 30-μm sections at −20 °C using a freezing microtome (NX50, Thermo Scientific, Waltham, MA, USA). For immunofluorescence staining, the sections were blocked in a solution of 5% normal donkey serum in 0.3% Triton-X in PBS at room temperature for 1 h. Subsequent to blocking, the sections were incubated at 4 °C overnight with primary antibodies against PINK1 (1:100), Iba-1 (1:100), Iba-1 (1:100), GFAP (1:100) and NeuN (1:100). Subsequently, the sections were subjected to appropriate fluorescently labeled secondary antibodies at 1:200 for 1h at room temperature. Nuclei were counterstained with DAPI.

Images were captured using a fluorescence microscope (VS120, Olympus, Tokyo, Japan) and analyzed with Image J 2 software (NIH, Bethesda, Rockville, MD, USA). Initially, we imported the fluorescence images, followed by separating the RGB channels and converting them into 8-bit grayscale images. Subsequently, we delineated the spinal dorsal horn region within the images, and we consistently applied a uniform threshold to standardize the process for quantifying the mean gray value. Notably, a minimum of three mice were included in each experimental group, with three randomly selected slices from each individual mouse were analyzed. Finally, average values were computed for each group to facilitate subsequent data analysis.

### 4.9. Western Blot Analysis

Under deep anesthesia, animals were sacrificed in order to harvest the L4-5 spinal cord segments. Tissue samples were collected and homogenized with RIPA buffer containing PMSF. The tissue samples were thoroughly mashed, and the protein extracts were obtained through centrifugation at 12,000× *g* for 15 min at 4 °C. The supernatants containing the protein extracts were collected. Protein concentrations were determined by the BCA assay, in accordance with the manufacturer’s instructions. Equivalent protein quantities from each sample were loaded and subjected to separation by SDS-PAGE gel electrophoresis. The proteins were then transferred onto PVDF membranes. To block non-specific binding sites, the membranes were blocked with 5% non-fat milk in PBST at room temperature for 1h to prevent nonspecific binding. Following this, the membranes were incubated overnight at 4 °C with appropriate primary antibodies against TNF-α (1:1000), IL-1β (1:1000), IL-6 (1:1000), PINK1 (1:250), Parkin (1:500), SQSTM1/p62 (1:1000), LC3B (1:1000), and β-actin (1:3000). The membranes were washed with PBST, followed by an incubation with appropriate secondary antibodies (goat anti-rabbit/mouse HRP) at a dilution of 1:5000 for 1h at room temperature. Protein bands were acquired using the ECL luminescence Reagent, and the images were captured by ChemiDoc Touch Imaging System (Bio-Rad, Hercules, CA, USA). The quantification of protein band intensities was performed using the Image J software.

### 4.10. RNA Sequencing

Three days after the CFA injection, mice were administered aucubin (10 mg/kg) for three consecutive days. Subsequently, the L4-5 spinal cord tissue was extracted. Three replicate samples were collected from each experimental group. RNA extraction was performed using the TransZol Up Plus RNA Kit reagents, followed by mRNA-seq analysis and data processing conducted by LC-Bio Technology Co. (Zhejiang, China).

### 4.11. Statistical Analyses

Statistical analyses were performed using GraphPad Prism 9.0 software (GraphPad, San Diego, CA, USA). All the data are expressed as mean ± SEM. For comparisons involving multiple groups, one-way or two-way analysis of variance (ANOVA) was performed, followed by appropriate post hoc tests to ascertain statistical significance. *p* < 0.05 was considered statistically significant.

## 5. Conclusions

In summary, our data demonstrate that mitochondrial dysfunction in mice with CFA-induced inflammatory pain. Aucubin exhibits potential as a candidate for alleviating inflammatory pain, likely attributed to its capacity to enhance autophagy, restore autophagic flux, and inhibit glial cell activation and pro-inflammatory cytokine expression. Consequently, our research sheds light on the crucial role of mitochondrial function in pain modulation, offering novel therapeutic avenues for pain management.

## Data Availability

Data is contained within the article.
